# Multi-organ Failure in a Patient With Chronic Ketamine Use: A Case Report

**DOI:** 10.7759/cureus.74158

**Published:** 2024-11-21

**Authors:** Ayoyimika O Okunlola, Syahmina Sufrian, Temitope O Ajao, G K M Rashik Uzzaman, Cornelius J Fernandez

**Affiliations:** 1 Internal Medicine, United Lincolnshire Hospitals NHS Trust, Lincolnshire, GBR; 2 General and Acute Medicine, United Lincolnshire Hospitals NHS Trust, Lincolnshire, GBR; 3 Diabetes and Endocrinology, United Lincolnshire Hospitals NHS Trust, Lincolnshire, GBR

**Keywords:** acute kidney injury, acute systolic dysfunction, gastric dilatation, hydronephrosis, ketamine, ketamine bladder syndrome, ketamine-induce cholestasis

## Abstract

Ketamine is increasingly used as a recreational drug. The report outlines the various multi-organ dysfunctions identified following chronic abuse in a 24-year-old patient with no significant past medical history and the subsequent patient management. Besides ketamine cessation, other treatments only provide suboptimal relief to the damage inflicted by the toxic ketamine metabolites.

## Introduction

Ketamine is a noncompetitive glutamate N-methyl-D-aspartate receptor (NMDAR) antagonist that causes muscle relaxation. It was initially developed as an anesthetic agent in 1963 [1]. Over the last 20 years, recreational use of ketamine has continued to rise among the young, predominantly male population, to induce a dissociative state and hallucinogenic experience [2,3,4]. Multiple organ damage involving the genitourinary system, hepatobiliary tract, gastrointestinal tract, heart, and brain have been identified in chronic ketamine users [1,3,5].

## Case presentation

A 24-year-old male became generally unwell following a recent use of 3 grams of ketamine about three days before presentation. He is known to abuse ketamine through sniffing for over one year, with abstinence of about 60 days before the current use. His past medical history involves anxiety and depression. He was under investigation for ongoing hematuria by the urology team and was planned for a flexible cystoscopy, which he failed to attend.

On admission, he appeared clinically cachectic, jaundiced, and dehydrated. His clinical observations at presentation based on the National Early Warning Score (NEWS) was five, comprising a pulse rate of 115 bpm, a respiratory rate of 26/min, a blood pressure of 139/97 mmHg, a temperature of 36.4°C, and oxygen saturation (SpO_2_) of 100% (on air). His venous blood gas (VBG) showed metabolic acidosis, and blood tests (Table 1) showed reduced renal function, severely deranged liver function, normocytic normochromic anemia, and raised inflammatory markers. The urine dip revealed hematuria. Besides this, blood culture yielded heavy growth of Streptococcus sanguinis. His toxicology screen for salicylate and paracetamol came back negative.

**Table 1:** Laboratory findings on initial investigation ALT: alanine aminotransferase; ALP: alkaline phosphatase; LDH: lactate dehydrogenase; INR: international normalised ratio; PT: prothrombin time; NT-proBNP: N-terminal pro-B-type natriuretic peptide; VBG: venous blood gas; PH: potential of hydrogen; GFR: glomerular filtration rate

Test	Result	Normal range
Sodium	129	133 – 146 mmol/L
Potassium	3.4	3.5 – 5.3 mmol/L
Bicarbonate	11	22 – 29 mmol/L
Urea	32.2	2.5 – 7.8 mmol/L
Creatinine	480	59 – 104 umol/L
Estimated GFR	14	90 – 200 mL/min
Bilirubin	64	0 – 21 umol/L
Direct bilirubin	58	1.7–5.1 umol/L
Indirect bilirubin	6.0	3.4–12.0 umol/L
ALT	268	0 – 41 U/L
ALP	645	30 – 130 U/L
Gamma GT	1928	10 – 71 U/L
Total protein	71	60 – 80 g/L
Albumin	23	35 – 50 g/L
C-reactive protein	322	0 – 5 mg/L
Hemoglobin	71	132 – 170 g/L
Mean cell volume (MCV)	81.0	80 -97 fL
Mean corpuscular hemoglobin (MCH)	29.5	26.9-33 pg
Mean corpuscular hemoglobin concentration (MCHC)	367	320-359 g/L
Haptoglobin	6.08	0.5 – 2.0 g/L
Reticulocyte	17.20	20 – 100 x 10^9/L
LDH	253	135 – 235 U/L
White blood cell count	12.8	4.3 – 11.2 x 10^9/L
Platelet	663	150 – 400 x 10^9/L
INR	1.3	0.9 – 1.1
PT	16.2	10 – 13
NT-proBNP	60904	0 – 300 ng/L
VBG results		
PH	7.310	7.320 - 7.430 kPa
Bicarbonate	13.1	22.0-29.0 mmol/L
Base excess	-12.1	2.0 mmol/L
Lactate	1.0	0.5 - 2.2 mmol/L

**Figure 1:**
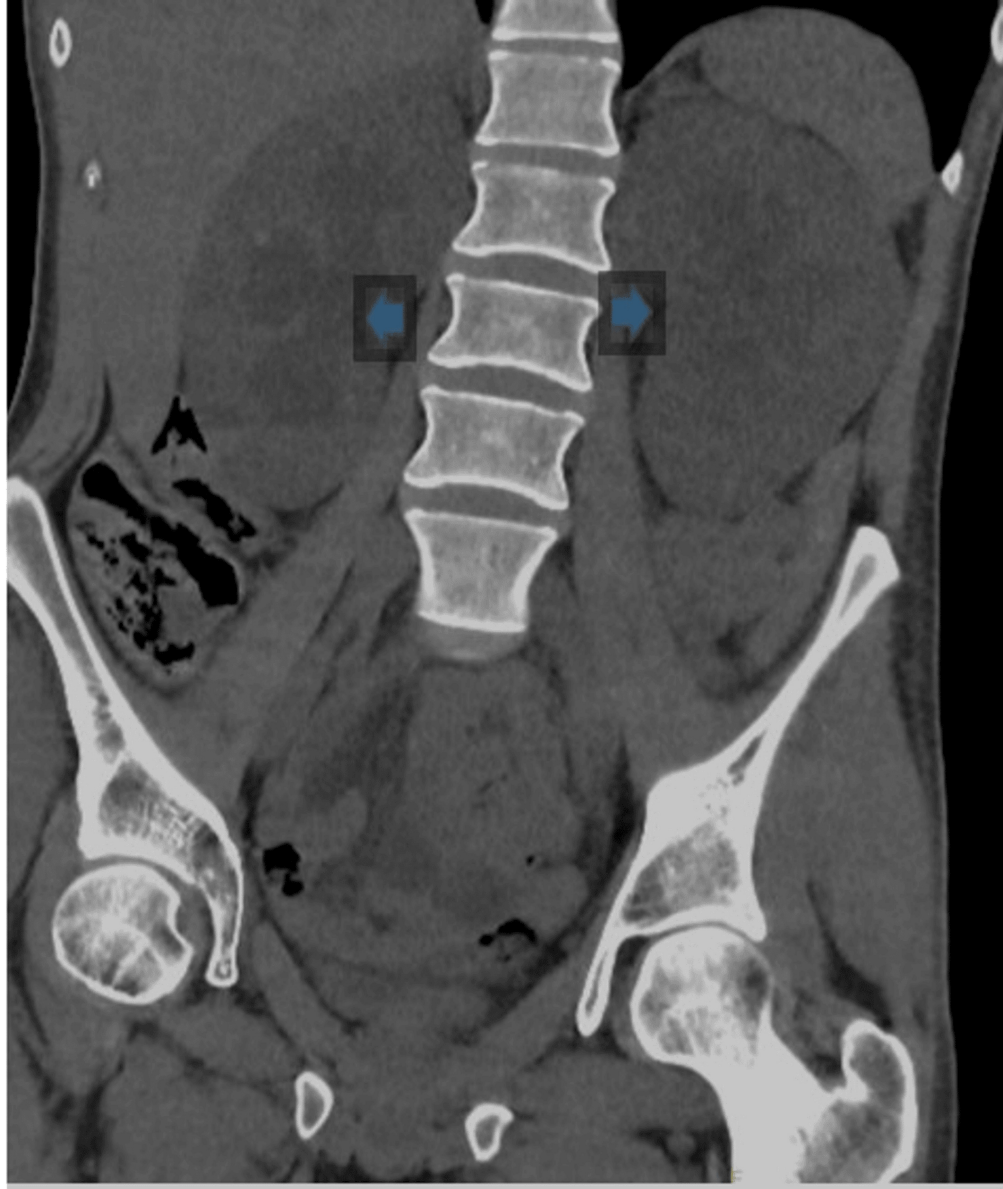
CT renal showing bilateral dilatation of pelvicalyceal system

**Figure 2:**
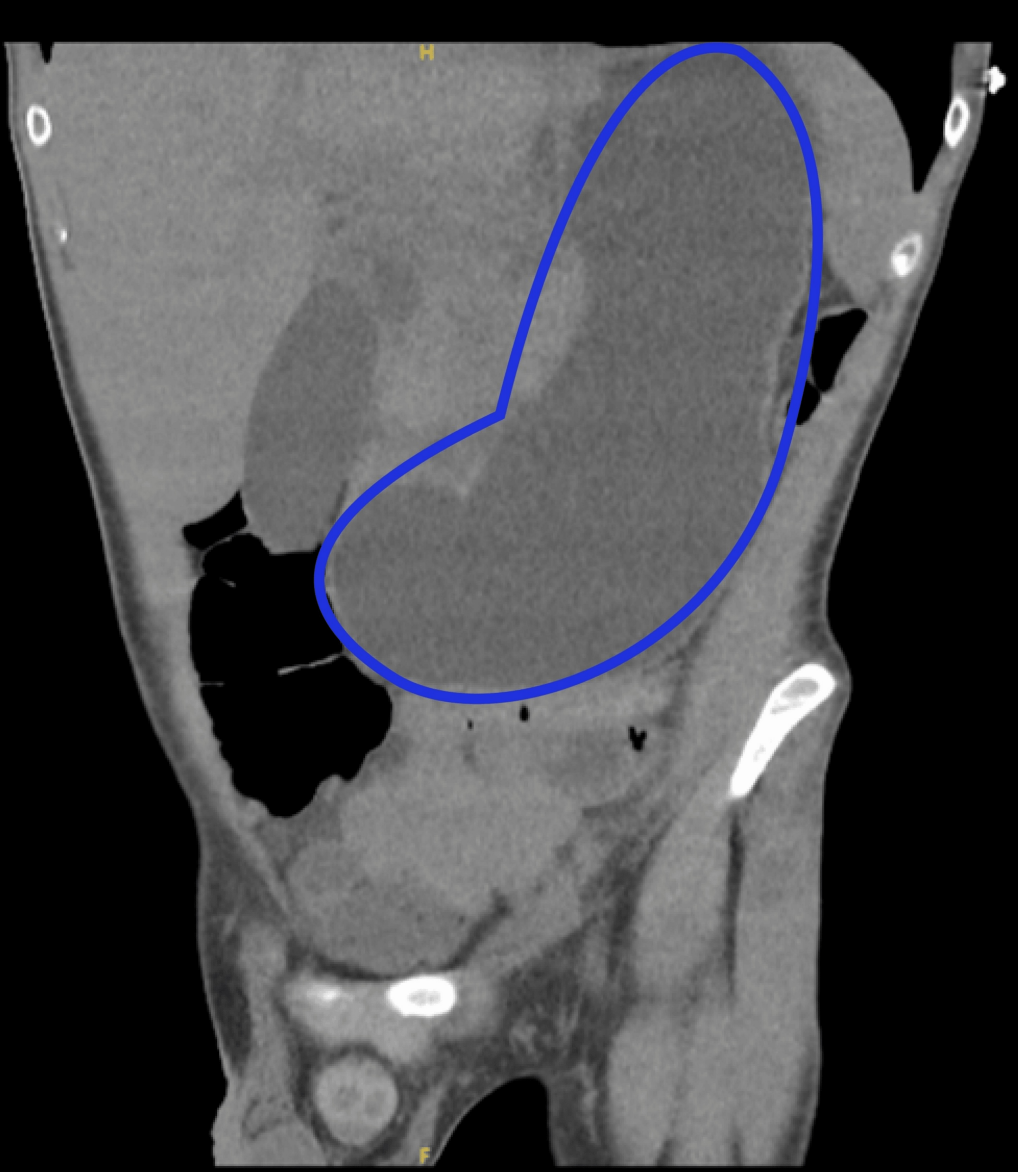
CT abdomen showing profound gastric dilatation

CT renal (Figure 1) demonstrated mild dilatation of the bilateral pelvicalyceal system (bilateral hydroureteronephrosis) with prominent ureters without obstructive ureteric calculus on either side. The urinary bladder was empty, and the stomach on CT abdomen (Figure 2) was profoundly dilated. Chest X-ray and CT head showed no significant findings. Ultrasonography of the abdomen has not shown any obvious pathology to explain cholestasis.

With the working diagnosis of sepsis and acute kidney injury, on a background of penicillin allergy, the patient commenced on intravenous ciprofloxacin. Upon arrival of a positive blood culture of Streptococcus sanguinis, which was sensitive to penicillin, discussion was held with the microbiologist, taking into cognizance the patient's allergy to penicillin. The antibiotic was changed to intravenous cefuroxime. He was catheterized at Accident and Emergency, and the urine was blood-stained. As the catheter caused significant pain and discomfort, the patient demanded it be taken out the next day. Repeated attempts to persuade him for re-catheterization failed.

Given the acute kidney injury and metabolic acidosis, nephrology advised intravenous bicarbonate and suggested focusing on relieving the obstruction via urgent nephrostomy. The patient eventually underwent a bilateral nephrostomy. The procedure was technically difficult on the left side due to ureteric fibrosis. His kidney function continuously improved, and he reached his baseline eGFR >90 on discharge. The urology team agreed with the diagnosis of ketamine bladder syndrome and scheduled an outpatient flexible cystoscopy, based on which to consider referring him to a tertiary center for radical cystectomy.

The hepatology team was also involved due to the markedly deranged liver function tests, and a diagnosis of ketamine-induced cholestasis was made. Other potential causes of deranged liver function, including autoimmune and infectious hepatitis, were ruled out by a non-invasive liver screen comprising of antinuclear antibody (ANA), smooth muscle antibody (anti-SMA), gastric parietal cell antibody, antimitochondrial antibody (AMA), liver kidney microsome antibody (anti-LKM), and hepatitis viral screening. The hepatology team expected that improvements in liver function would happen upon ketamine abstinence.

**Figure 3:**
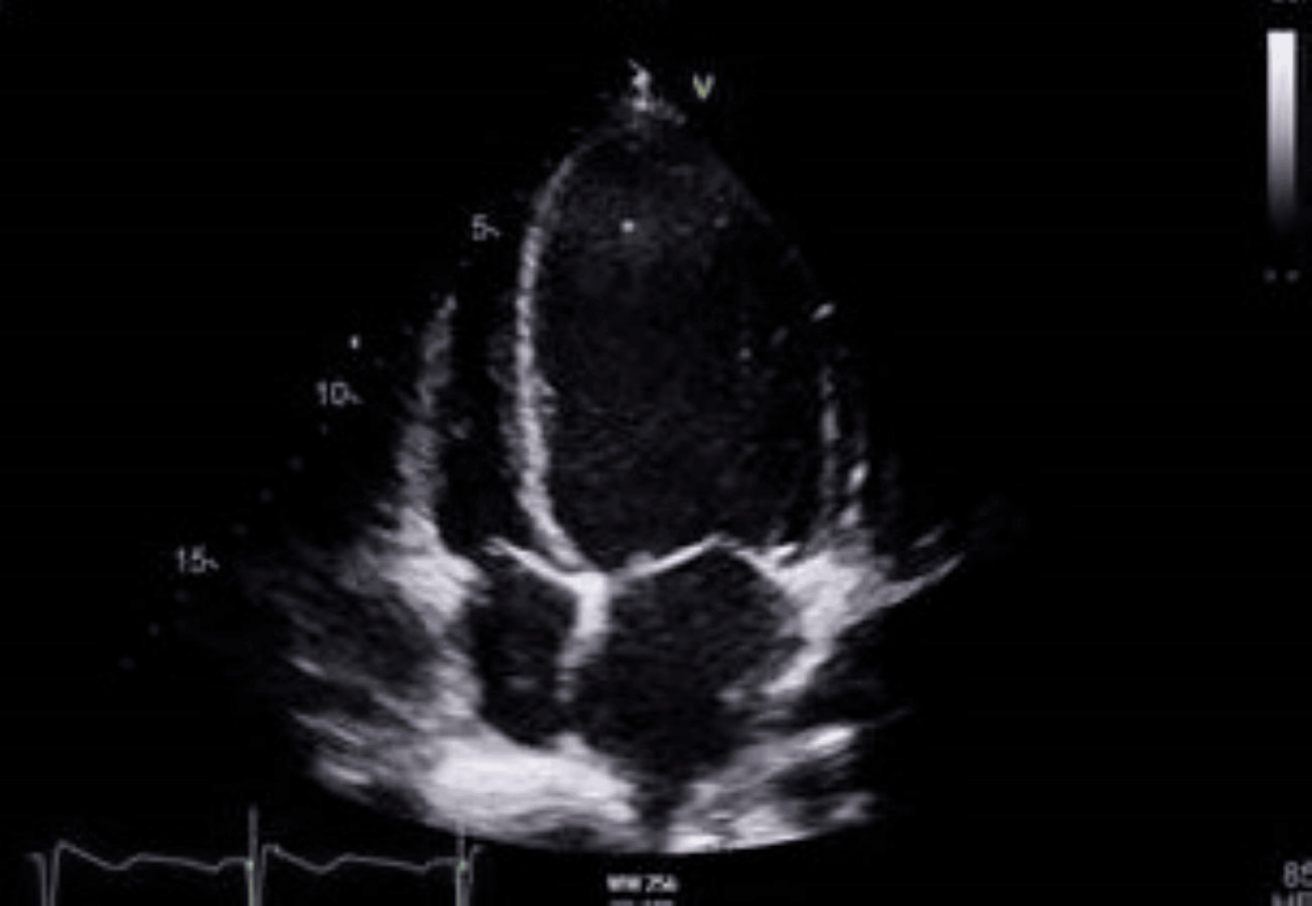
Transthoracic echocardiogram showing slightly dilated right atrium

**Figure 4:**
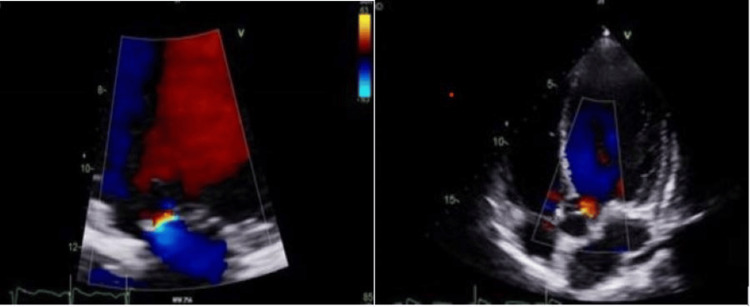
Transthoracic echocardiogram a: trace mitral regurgitation; b: trace aortic regurgitation

A transthoracic echocardiogram (Figure 3, 4) was performed due to Streptococcus sanguinis bacteremia. The echocardiogram showed impaired left ventricular systolic function, with an ejection fraction of 37%, grade I diastolic dysfunction, dilated left atrium, and borderline enlargement of the right atrium with trace aortic and mitral regurgitation without any evidence of endocarditis. His N-terminal pro-B-type natriuretic peptide (NT-proBNP) was raised, and disease-modifying heart failure medications (bisoprolol, sacubitril/valsartan, and spironolactone) were added in a stepwise manner once the acute kidney injury resolved. The heart failure team plans to start him on sodium-glucose cotransporter-2 (SGLT-2) inhibitor once the bladder issue resolves, after having undergone radical cystectomy.

The full blood count (Table 1) showed a normochromic normocytic picture with thrombocytosis. Blood film showed a mixed picture of target cells, fragmented red blood cells, elliptocytosis, poikilocytosis, and hypochromia. Initial concerns of autoimmune hemolytic anemia were allayed by the hematologist after the following investigations: negative Coombs test, normal reticulocyte count, haptoglobin, and lactate dehydrogenase (LDH). The hematologist concluded that anemia may be due to liver derangement and thrombocytosis may be due to infection.

## Discussion

Chronic ketamine abuse can cause multi-organ dysfunction. Urinary tract abnormalities are the most common features among chronic abusers. At least 26-30% of chronic ketamine users experience ketamine bladder characterized by ulcers, cystitis, and fibrosis, leading to incontinence, hematuria, overactive or shrunken bladder, and in advanced stages, hydroureter and hydronephrosis, depending on dose and frequency of use [4]. Ketamine or its metabolites are thought to cause a direct cytotoxic insult to the lower urinary tract, resulting in inflammation, ulceration, and varying degrees of damage [3,6]. A flexible cystoscopy provides a direct visualization, which may include varying severity of glomerular hemorrhage, mucosal edema, and ulceration [7]. No definitive treatment has been identified yet for ketamine bladder, except abstinence, and damage is reported to be irreversible [3,6].

Currently, reconstructive surgery through cystectomy is deemed partially effective [7,8,9]. A recent case study in 2023 [7] documented that in post-reconstruction, the majority developed complications requiring further intervention, although the ketamine use status of these patients is unclear. This case study also provided a helpful outline of the treatment approach, starting with symptomatic relief consisting of oral anti-inflammatory drugs and pentosan polysulfate, then intravesical treatment, and ultimately surgical intervention in the form of reconstruction or cystectomy [7]. Considering the young age of this patient, reconstructive surgery could still yield benefits as it would give him the best chance at symptom alleviation, which subsequently offers a better quality of life. In addition, ketamine cessation support would be essential given a possible relapse of abuse.

Kidney injury secondary to chronic ketamine abuse can be a result of its paralytic effect causing vesicoureteral reflux, either unilaterally or bilaterally [8]. The vesicoureteral reflux causes upstream pressure leading to hydroureter and hydronephrosis, as seen in CT findings (Figure 1). Decompression through bilateral nephrostomy could improve the estimated glomerular filtration rate (eGFR). However, a longitudinal follow-up study reported that about 68% of those with ketamine-induced hydronephrosis displayed a gradual deterioration in kidney functions, despite optimal interventions [10]. These advanced ketamine bladder patients are five times more likely to experience deterioration in renal function compared to those with isolated cystitis, with a minority ending in end-stage kidney failure requiring hemodialysis [10]. Close monitoring should be offered to all patients with ketamine-induced hydronephrosis, due to the inevitable irreversible damage that can occur.

Acute interstitial nephritis from ketamine use has been shown in all cases in a rat model, with persistent interstitial nephritis in over 60% of the rats following abstinence [11]. The first case of acute interstitial nephritis in humans severe enough to require hemodialysis was reported in 2022 after repeated ketamine infusions [12]. As biopsy is not routinely done, it is difficult to predict how common acute interstitial nephritis in ketamine users is.

Cholestasis is common among chronic ketamine abusers [1,10,13]. The levels of alkaline phosphatase (ALP) and gamma-glutamyl transferase (GGT) are claimed to be linked to the severity of ketamine-associated uropathy [10]. Ketamine undergoes extensive hepatic metabolism, primarily through the cytochrome P450 system (CYP 3A4), which could explain this liver enzyme derangement. Liver biopsy may reveal common bile duct dilatation, microscopic bile duct injury, and liver fibrosis [13]. The mechanism by which ketamine causes biliary abnormalities remains unknown. However, the high concentrations of ketamine metabolites in the bile may lead to direct toxic injury to the surface epithelium with repeated use [9]. Full recovery from the hepatobiliary disease over time has been observed with complete abstinence from ketamine abuse [14].

Epigastric pain, nausea, and vomiting are the commonest gastrointestinal presentations in individuals with chronic use of ketamine, accounting for up to 75% of symptoms [3]. Gastric dilatation (Figure 1) could be attributed to gastric dysmotility leading to delayed emptying. This gastric paralytic effect of ketamine is also seen in another case report [6]. The cachexia seen in our patient resembles that of a malignancy. This could be attributed to upper gastrointestinal disturbance and the resulting poor nutrition. In addition, biliary dysfunction and acute kidney injury might also contribute to this. Previous case reports demonstrated endoscopic evidence of esophagitis and gastritis in chronic ketamine abusers [3].

Ketamine use disorder can lead to severe cardiovascular complications, including acute systolic heart failure, likely due to its direct negative inotropic effects [15]. Though our patient exhibited heart failure with reduced ejection fraction (HFrEF) based on echocardiogram and NT-proBNP, he remained asymptomatic for heart failure throughout hospitalization. Cardiopulmonary toxicity of ketamine is significantly rarer compared to other systemic effects. A few cases of stress cardiomyopathy caused by preoperative ketamine administration have been reported [16]. Two case reports have identified a connection between myocardial fibrosis and chronic ketamine use [17]. Saliba and Mina recommend that healthcare providers should consider screening for ketamine use disorder in young adults who present with acute systolic heart failure [15].

Degenerative changes in different parts of the brain on magnetic resonance imaging have been documented as hyperintense spots in the cerebral cortex, internal capsule, basal forebrain, cerebellum, pons, and diencephalon depending on the chronicity of ketamine abuse [5]. The structural damage on neuronal and synaptic levels is evident within one year of abuse in individuals with no prior neurological disease [5]. Although not observed in this case, chronic abuse can present with a range of neurological symptoms such as cognitive disturbance, mood disorders, and psychotic and dissociative symptoms [3,9,18].

This case demonstrates multiple organ dysfunction secondary to ketamine abuse, as evidenced by acute kidney injury, ketamine bladder-causing hydroureter, hydronephrosis, hematuria, cholestasis, gastric dilatation, and left ventricular systolic dysfunction. His successful management involved a multidisciplinary approach with inputs from specialties including nephrology, urology, hepatology, cardiology, hematology, and microbiology.

## Conclusions

Chronic ketamine abuse can have a multi-system effect, ranging from well-documented effects on the lower urinary tract with subsequent renal injury and cholestatic liver changes to rather rare neurological and cardiotoxic effects. Regardless of the underlying mechanism, ketamine cessation is the cornerstone of successful treatment.
